# Overwintering covered with soil or avoiding burial of wine grapes under cold stress: Chinese wine industry's past and future, challenges and opportunities

**DOI:** 10.1007/s44154-023-00119-9

**Published:** 2023-09-15

**Authors:** Ningjing Wan, Bohan Yang, Dingze Yin, Tingting Ma, Yulin Fang, Xiangyu Sun

**Affiliations:** 1 College of Enology, Shaanxi Provincial Key Laboratory of Viti-Viniculture, Viti-Viniculture Engineering Technology Center of State Forestry and Grassland Administration, Shaanxi Engineering Research Center for Viti-Viniculture, Heyang Viti-Viniculture Station, Ningxia Eastern Foot of Helan Mountain Wine Station, Northwest A&F University, Yangling, 712100 China; 2 Center for Viticulture and Enology, School of Agriculture and Biology, Shanghai Jiao Tong University, Shanghai, 200240 China; 3 College of Food Science and Engineering, Northwest A&F University, Yangling, 712100 China

**Keywords:** Wine grape, Northwest China, Cold stress, Cold-resistant culture, Bury and unearth vines, Overwinter safely

## Abstract

In northwest China, where winter is extremely cold and the grapevine is vulnerable to freezing damage, the application of soil covering has promoted the vigorous development of the local grape and wine industries. However, in recent years, the negative effects of burying soil for cold protection on the environment have gradually emerged. In some viticultural regions, the phenomenon of "summer forest, winter desert" has appeared. Therefore, it is urgent for the Chinese grape industry to find a better solution to overwinter safely and environmentally friendly. This review summarizes the advantages and disadvantages of widely used solutions to overwinter such as covering vines with soil, breeding of cold-resistant grapes, cold-resistant cultivation model, physical and chemical covering materials, and protected grape facilities were reviewed. Future overwintering measures were proposed which avoid burial and grape overwintering research directions. It also provides a theoretical foundation and technical support to improve grape yield and quality in northwest China.

## Introduction

Traditional wine regions are mostly Mediterranean with mild and rainy winters, which meet the minimum night temperature (above 0 °C) required for grape survival (Hannah et al. 2013; Wang et al. 2018). Even Germany, the northernmost wine region in the world, does not require soil-burying to safely over-winter. First of all, the winter temperature in Germany is about 0℃, and the extreme minimum temperature is about minus 10℃, which does not reach the lethal temperature of the vine. Secondly, the regulating effect of the Rhine, Mosel and Main rivers on temperature can also reduce the damage caused by winter frosts. In addition, the vines are cold-resistant varieties suitable for local conditions, such as *Riesling* and *Vidal*. Finally, occasionally, when extremely low temperatures occur, irrigation, torches, and blast blowers are applied in the vineyard to keep the vines warm. However, the average winter temperature in most grape-producing areas in northern China is less than -15 °C. Under freezing stress during dormant period, grapevine and root system are prone to frostbite. Intracellular ice formation leads to cell death, and extracellular ice formation provokes osmotic stress. This osmotic stress mainly causes changes in lipid polymorphism, expansion-induced lysis, which leads to cell death, and increased ROS production (Ambroise et al. 2020). This causes late or no germination of vines the following year, which leads to the deterioration of grape berry quality and even the death of the whole plant (Horiuchi et al. 2021). Therefore, in the twentieth century, severe winter brought great challenges to the development of the Chinese grape industry, and it was widely believed that China could not grow grapes on a large scale. Since the 1950s, the technology of covering vines with soil for winterization has been applied on a large scale in northwest China (Lu et al. 2020), and Chinese viticulture and wine industry has developed on a large scale. Currently, the relatively high-quality viticulture areas are mainly distributed in the northwest region, which needs to covering vines with soil (Xue et al. 2019).

**Fig. 1:**
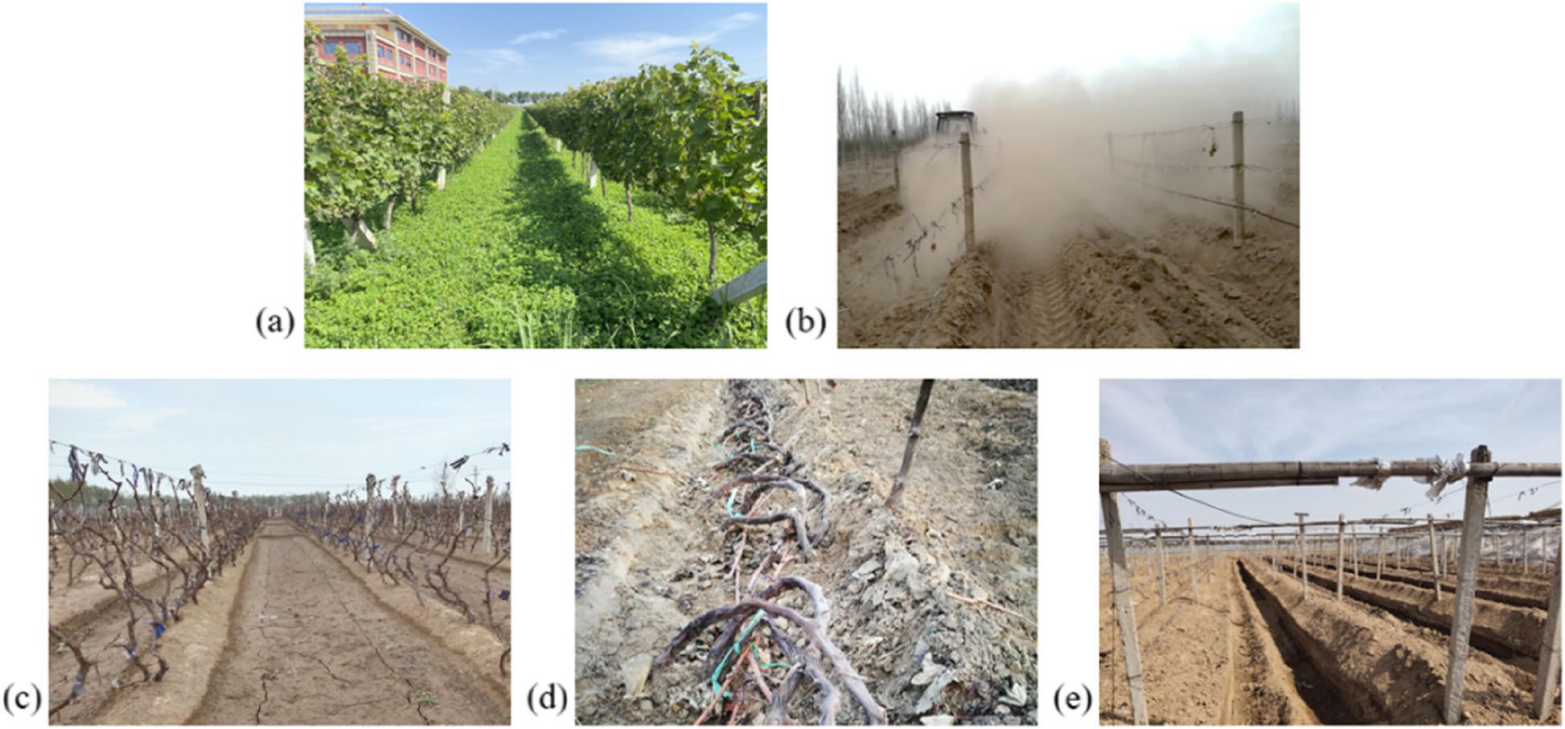
Seasonal changes and steps of winterization scene in vineyard. **a** Forest in summer; **b** Desert in winter;** c **Vineyard before covering with soil;** d **Remove vine branches from the rack;** e **Vineyard after covering with soil

However, as time and area increased, the environmental damage gradually became apparent. The phenomenon of "summer forest, winter desert" in the grape growing area (Fig. 1a) damaged the soil surface, accelerated wind erosion and soil carbon emission (Wang et al. 2015). Additionally, due to the shortage of manual labour in agriculture, labor costs increase greatly. At the same time, covering vines with soil accelerated plant senescence, reduced grape quality, and reduced the efficiency of mechanized production (Novara et al. 2018). Inevitable mechanical damage during burial can distort individual cells, leading to the extension of the cell wall and eventual breakage (Ma et al. 2023). Cell breakage can stimulate the production of large amounts of reactive oxygen species (ROS), leading to significant levels of membrane lipid peroxidation and increasing the activities of phospholipase D (PLD), lipase, and lipoxygenase (LOX), thus reducing cell membrane integrity (Boava et al., 2011; Li et al. 2017; Reymond et al. 2000). Besides, wounds caused by mechanical injury are potential infection sites for pathogens. In order to cope with microorganism infections, plants typically induce the expression of numerous defense genes at the injured site in response to mechanical injury, such as plant pathogen-related (PR) proteins (POD, PPO, β-1,3-glucanase, PAL, chitinase, etc.) (Gu et al. 2022).

This technology cannot meet the needs of the the development of Chinese grape industry. China is in urgent need of an eco-friendly and mechanized method to replace it (Zhou et al. 2021a, b, c). The review summarized the research at home and abroad in terms of the advantages and disadvantages of cold prevention measures, such as covering vines with soil, breeding cold resistant grapes, cold-resistant cultivation model, physical and chemical covering materials, and protected grape facilities facilities. The main measures and the research direction for grape overwintering are forecasted in the future.

## Covering vines with soil to prevent cold

### Method of covering vines with soil

The method (Fig. 1b, c) is to dig the soil between grape rows to cover the vine after removing the vines from the rack and placing them on the ground. At present, it is a method widely used in production. It is generally believed that the isotherm of -17 °C in winter is the dividing line between burying soil for winterization and overwintering in open land (Han et al. 2014).

Different climates in different places lead to different periods of cold protection. Generally, it is 10 to 15 days before the soil freezes. In spring, when the soil defrosts and the temperature reaches about 10 °C, the vines are placed on the shelf to ensure that the buds are intact and not mouldy. Also, the buried width of the soil should be 1 m. The soil cover should be more than 30 cm in the branches, and the digging should be more than 80 cm away from the trunk.

### Advantages of covering vines with soil

According to the international wine grape climate regionalisation index system, the southeast of China is a suitable area for wine grapes (Du et al. 2022), but in fact, due to the high temperatures and rainy summer, the growth of wine grapes was poor (Li et al. 2011a, b). At the same time, the average temperature in most winters in northern China is lower than -15 °C, which easily causes freezing of grape roots and even the death of the entire plant. Therefore, in the twentieth century, severe winter brought great challenges to the development of the Chinese grape industry, and it was generally believed that large-scale grape planting could not be done in China.

**Fig. 2:**
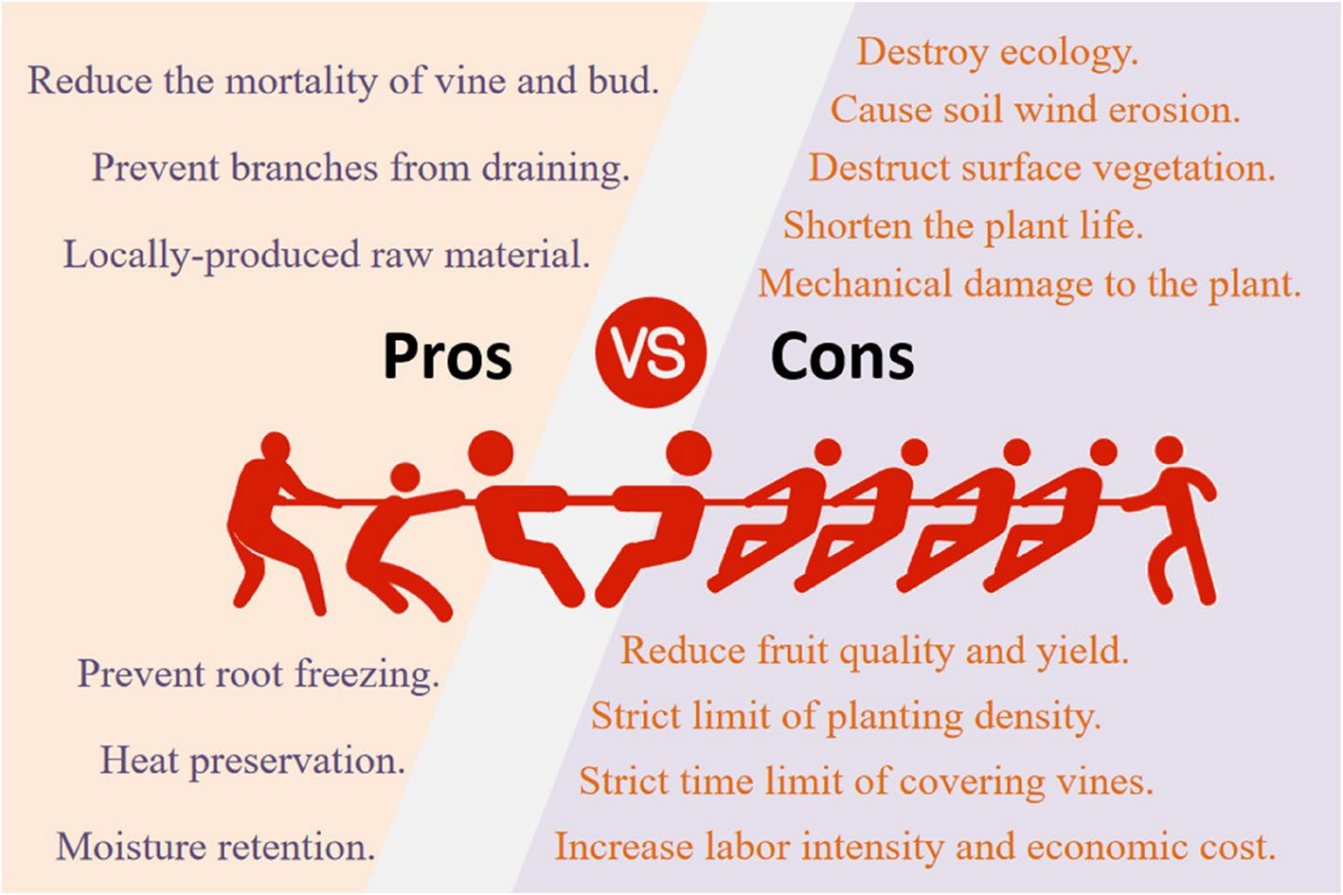
The pros and cons of covering vines with soil in winter

Since the widespread application of the technique in the 1950s, vines can be insulated and moisturised throughout the winter, effectively preventing the branches from drying out and the roots from freezing (Fig. 2). After this technology was widely applied in China, Li Hua re-established that most of northwest China was suitable for wine grape cultivation through grape climatic regionalization research (Li et al. 2011a, b). The wine grape industry is developing rapidly in China and has become the largest wine grape growing area in the world. By 2022, the grape planting area have reached 785 kha (OIV 2022).

### Disadvantages of covering vines with soil

#### Environmental effects

As time and area of the technology increased, the damage to the ecological environment gradually increased. About 87.5% of the Chinese wine grape cultivation area is in areas where vines need to be buried (Xie 2018), and the grape producing areas are mostly distributed in the arid and semi-arid areas with fragile ecology. Vegetation will be destroyed during soil burying, which can cause up to six months of soil exposure, as a result of the phenomenon of "summer forest, winter desert" in the grape growing area. Wind erosion is evident in bare soil, easy to cause soil surface damage, has negative impacts on ecology, and is not conducive to environmental protection (Fig. 2).

#### Rising costs and labour shortages

Compared to Old World wine regions, 90% of grape varieties in China are buried to ensure safe overwintering. It is labor-intensive which generates a lot of extra costs each year, including labour, facilities, and operation expenses. More than one third of the cost of viticulture in northern China is used to cover the soil in winter and unearthing vines in spring. The annual cost of burying soil and removing vines even exceeds ¥15,000/ha in Xinjiang. Taking winter of 2018–2019 as an example, each bottle of wine costs ¥2.93 more than it does in the Old World wine countries, where winters are mild (Liu et al. 2021).

Due to the fact that burying soil and unearthing vines require heavy workers, manual burial is inefficient, time consuming, and easy to damage vines. Recently, the work has become semi-mechanized and mechanized. Grape vine burying machines are easy to ditch, can replace 7–8 labours, save costs, and greatly improve the efficiency of grape production. Furthermore, the germination and growth of the vines will be better than those of the artificially buried vines in the next year. However, the existing grape vine burying machine has poor soil crushing effect, a close distance for throwing soil, low efficiency of operation, a large volume, and a large amount of turning space (She 2017). Furthermore, it requires fixed row spacing between vines, which has higher requirements for standardized construction of vineyards, and it needs to carry out adaptive planning during vineyard construction, which is difficult to promote in the established vineyards (Yang et al. 2018).

The existing grapevine soil cleaner mainly uses a brush or scraper to remove the soil attached near the grapevine. This method significantly reduces labor cost, improves the excavation efficiency, and provides the possibility for the mechanised planting of wine grapes. However, discovering grapevines is a delicate process, and the distribution in soil ridges is complex. As a result, the soil cleaner is easy to destroy the branches and rip off the expanded buds. Moreover, the soil cleaner can only remove the outer layer of soil on both sides and the top and cannot remove all the soil cover in the central area of the grape. The residual soil still needs to be removed manually. To achieve full soil cleaning effect, a wine-grape noncontact blower that can be hung on a tractor and operated while driving can use a scraper to remove 60% of the cold-proof soil and then use a blower to blow away the remaining soil (Yang et al. 2021). However, this method will cause dust to blow across the sky in the process of operation, affecting the ecological environment and human health.

Our grape planting range is broad, planting row spacing varies, but operation width is typically less than 2.5 m. Therefore, large-scale vine burying machines are restricted, and small and medium-sized vine burying machines are the development direction. At the same time, researchers in the future should focus on improving the mechanization in vineyards, which can optimise fertilizer application methods, thus reducing fertilizer input and preventing overfertilization and pesticide use in vineyards (Yang et al. 2022). In addition, smart agricultural applications can also help reduce the use of pesticides and fertilisers in grape production by reducing energy consumption (Akdemir 2022).

## Research progress of overwintering measures which avoiding burial

**Fig. 3:**
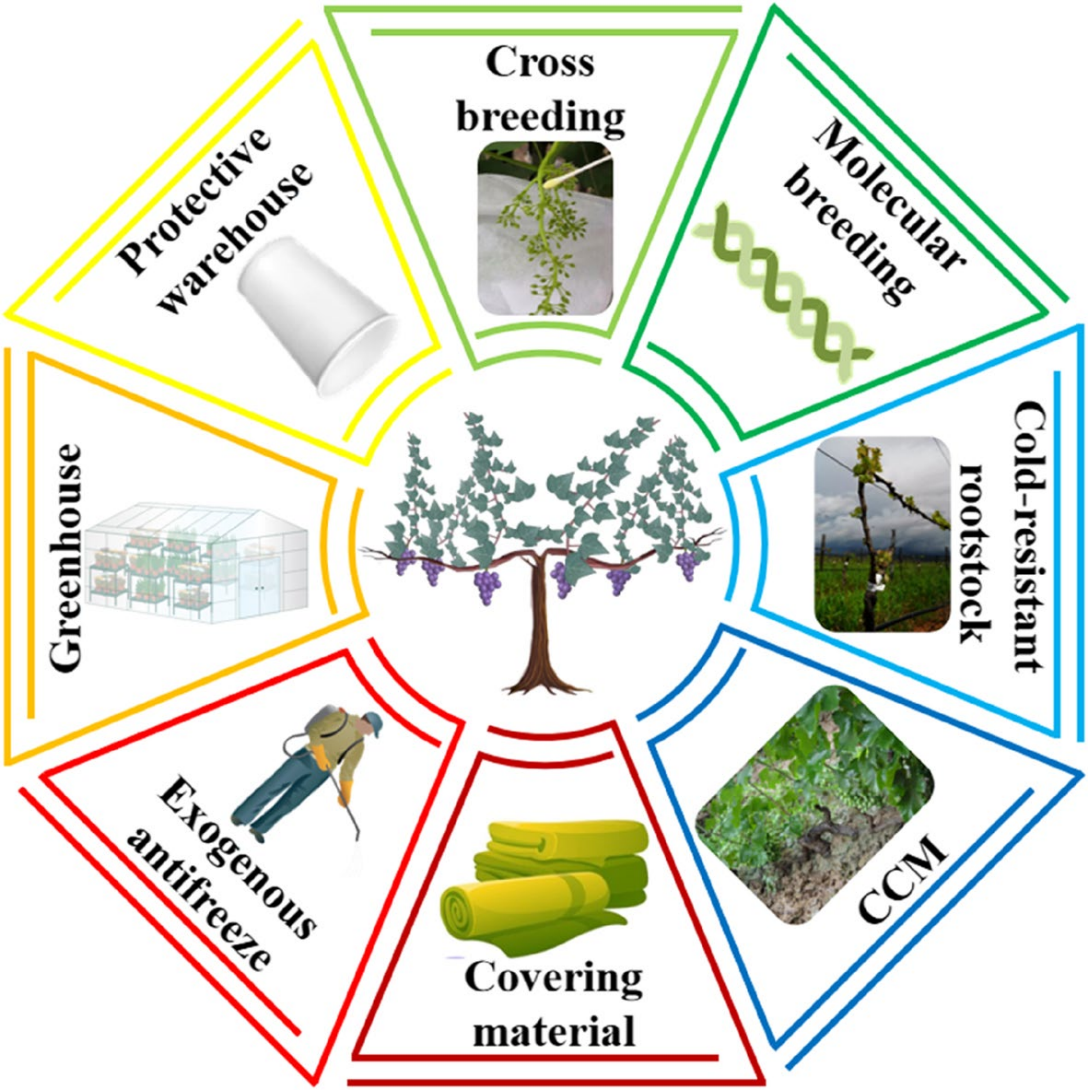
Eight overwintering measures which avoid burial

Overwintering measures are directly related to the growth and quality of the next year's grapes. In addition, it will affect the long-term plan of vineyard development. Compared to the current method of covering vines with soil, overwintering measures that avoid burial can reduce the cost and the demand for labour, which is conducive to improving the economic benefits of grape production (Han et al. 2021). At the same time, damage to soil surface can be avoided. As a result, it is necessary to investigate the overwintering measures that avoid burial in China, which can be used as a future viticulture model in areas where grapes need to be buried. In the long run, the fundamental approach is to breed varieties with cold resistance and good wine quality according to the requirements of industrialisation. However, due to the lengthy breeding cycle, the key at this time is to improve the cultivation method by using cold-resistant rootstock grafting seedlings. At the same time, it is also an effective way to choose cost-effective covering materials and adopt grape-protected facilities. The following four aspects of the measures are introduced: breeding, cultivation model, covering materials, and protected grape facilities (Fig. 3), which are representative of the overwintering measures available in Chinese vineyard cultivation and management of Chinese vineyards.

### Breeding of cold-resistant grapes

The breeding of grapes for cold resistance is the key to further development of the grape industry in cold regions (Wang et al. 2021a, b). The basic method for cold-resistant grape production and cultivation is to cultivate a dormant tree body whose germination nutrition and reproductive organs are resistant to frost and low temperature, and then produce new varieties of high-quality wine grape (Zhang et al. 2018). To improve the overwintering ability of grapes, breeders developed varieties adapted to different regions with early fruits, fast branch ripening, and late stages of germination. Only by establishing commercial vineyards under the premise of grape breeding can we produce high-quality grape fruits. The excellent grape varieties show high yield, stable yield, high quality, low consumption, strong resistance to stress, good adaptability, and have good promotion and utilisation values in production. They can therefore obtain better economic benefits.

#### Cross-breeding

**Fig. 4:**
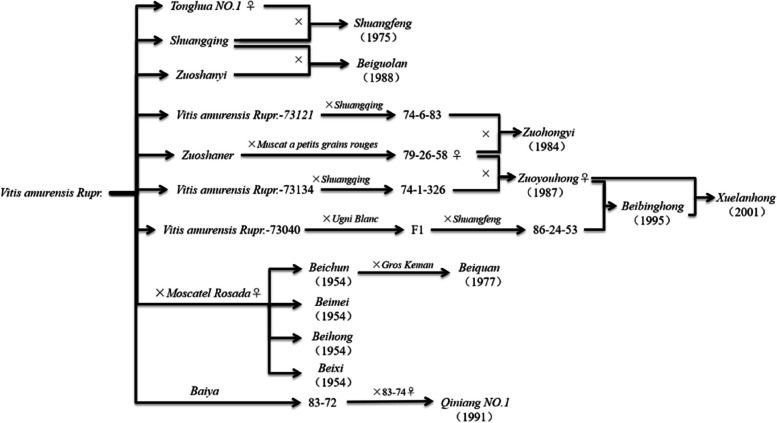
Seedlings and hybridization of cold-resistant varieties produced from *Vitis amurensis*

Cross-breeding is based on the principle of gene recombination. Parents with desired traits are selected for hybridisation, and progeny with good traits are continuously selected for self-cross, backcross or hybridization, and finally offspring with a variety of good-character germplasm aggregation are bred. In the past 70 years, China has made unremitting exploration and research on cold-resistant grape breeding and achieved some achievements. *Vitis amurensis Rupr.* is considered one of the most cold-resistant grape varieties, capable of withstand temperatures as low as -40 °C (Wan et al. 2008; Gu et al. 2020). Currently, the cold resistant germplasm of grapes has been widely excavated and utilized in China (Chai et al. 2019) (Fig. 4).

Institute of Botany, the Chinese Academy of Sciences, hybrid bred the *Beifeng* (Fan et al. 2007), the *Beizi* (Fan et al. 2006) by *Vitis bryoniifolia Bunge* and *Moscatel Rosada* in 1953, hybrid bred the *Beichun* (Li et al. 1983), *Beihong* (Jiao et al. 2022), *Beimei* (Wang et al. 2009) and *Beixi* (Wang et al. 2014a, b) by *Moscatel Rosada* and *Vitis amurensis Rupr.* in 1954, bred the *Beiquan* in 1977. In 2004, *Xinbeichun* was selected by the bud mutation of the *Beichun*. These cultivars are highly resistant to cold, and do not need to be buried during winter in North China, and they have been extended to about 1,000 ha (Wang et al. 2020a, b). Among them, *Beichun* has been cultivated in Henan, *Beimei* and *Beihong* have been introduced and cultivated in Tianjin, Zhejiang, Ningxia, Xinjiang, Gansu, Guizhou, Inner Mongolia, and Guangxi (Zhou et al. 2021a, b, c). *Beihong* produces a wine with blueberry and plum aromas; *Beimei* produces a musky, full-bodied wine. However, the quality of their wines is unstable. In Ningxia, the single-variety wine has high acidity, poor palatability and unbalanced body (Jiao et al. 2022).

The Liaoning Institute of saline-alkali Land Utilization cultivated *Zhuosexiang* in 1961. It is the main variety of greenhouse in northern China. The grape skin is purplish red, sweet taste, and has rich scent of jasmine (Li 2021). The grape has the characteristics of early maturity, low temperature resistance, dense planting resistance, good flower bud differentiation, extensive management, and high market price. It has been widely promoted, including in Liaoning, Jilin, Inner Mongolia, Hebei, Henan, Yunnan, etc.

The Institute of Special Animal and Plant Sciences CAAS produced *Shuang Feng* (1975), *Shuanghong *(1977), *Zuohongyi* (1984) (Lu et al. 2000), *Zuoyouhong* (1987) (Song et al. 2005), *Beiguolan* (1988) (Zhang and Lu 2016), *Beibinghong *(1995), and *Xuelanhong* (2001) (Song et al. 2012). At present, *Shuangqing*, *Shuangfeng,* and *Zuohongyi* are gradually phased out due to low wine yield, poor quality, and, most importantly, weak resistance to downy mildew (Liu and Li 2013). *Zuo Youhong* has a small viticultural area and is mainly used to make dry red wine. *Xuelanhong* is the sixth generation of *Vitis amurensis Rupr.*, and its cold resistance is similar to that of *Vitisriparia* × *V.labrusca*. It can overwinter in open land in northeast China. At the same time, *Xuelanhong* is resistant to disease, has a large panicle, a high yield, a high sugar and juice yield, low total acid and tannin, and can produce high-quality dry red wine (Wang et al. 2014a, b). In the northeast, *Shuanghong* and *Zuoyouhong* are the main varieties. *Beibinghong* is cultivated in Hebei, Northeast and Shaanxi.

*Qiniang No.1* was bred by the Horticulture Research Institute of Qiqihar, Heilongjiang Province, in 1991 (Bi et al. 2014). It has strong cold resistance and can be overwintered simply by covering 10 cm of soil, and there is no freezing damage to branches or buds. However, in winter drought, it is impossible to winter without covering the soil. When winter protection, pay attention to winter water replenishment in drought years, and plastic film can also be covered in cold prevention to maintain humidity and prevent branches from draining.

In addition to *Vitis amurensis Rupr.*, Northwest A&F University crossbred *Meili* by *Merlot*, *Riesling*, and *Moscatel Rosada* in 1982 (Zhang et al. 2013a, b) and crossbred *Ecolly* by *Chenin blanc*, *Chardonnay*, and *Riesling* in 1983. *Meili* dry red wine is ruby red in colour with fruity aromas of wine aromas that are mellow and harmonious (Meng et al. 2017). *Ecolly* is strong resistant to disease, cold, and drought. Its aroma of dry white wine is pure, elegant, mellow, and soft; and its body is balanced. They are mainly grown in Shanxi and Shaanxi. In addition, the Meili series is one of the main products of Inner Mongolia Sunshine Tianyu International Winery.

#### Molecular breeding

Most traditional breeding varieties gain cold tolerance at the expense of quality or yield and thus cannot meet the needs of modern society. It is necessary to seek new methods, such as molecular breeding, to breed hardy varieties. Currently, the most important technique in molecular breeding is Marker-Assisted Selection (MAS) (Devi et al. 2017). The principle is that after hybridisation with resistant parents, molecular markers closely linked to resistant genes are screened according to gene linkage separation and tested in the progeny. Thus, as long as the single plant itself carries a specific molecular marker, it is considered to carry the resistance gene. This method can be tested at the planting stage or seedling stage, improving the precision and efficiency of breeding. At the same time, minor-polygenes substitution and accumulation (MPSA) and intraspecific recurrent selection in *V. vinifera* (IRSV) can also be used for breeding cold-resistant grapes and other desirable cold-resistant traits to improve the quality and disease resistance of grape varieties (Han and Wang, 2021).

So far, researchers have cloned several genes in mountain grapes, such as *VaERF080*, *VaERF087*, *VaCPK20*, *VaCBF1*, *VvCBF4*, *VaICE1*, *VaSAP15*, *VvBAP1 and VaMYC2*, which play an important role in the tolerance of plants to cold. Both *VaERF080* and *VaERF087* can improve the cold tolerance of transgenic Arabidopsis Thaliana by increasing the activity of antioxidant enzymes and regulating the expression of cold-related genes (Sun et al. 2019). Overexpression of *VaCPK20* increased the expression of stress-responsive genes (such as *COR47*, *NHX1*, *KIN1*, or *ABF3*) in transgenic Arabidopsis thaliana plants without stress, after freezing, and under drought stress (Dubrovina et al. 2015). The *C-repeat binding factor* (*CBF*) was found to be an important transcription factor for plant resistance to stress in Arabidopsis thaliana. Furthermore, the *VaCBF1* and *VvCBF4* genes were isolated from riverbank grape and mountain grape and proved to improve the freezing resistance of wine grape (Aazami and Mahna 2017; Xv et al. 2021). *VaICE1* acts upstream of *CBF* in the cold stress pathway, binding and activating the *CBF3* promoter, which subsequently binds to the *CRT/DRE* cis-acting element (*CCGAC*) in the promoter region and induces the expression of the downstream cold response gene (*COR15A*) and other cold adaptation genes, thus improving frost resistance (Dong et al. 2013). The overexpression of the *VaSAP15* gene isolated from highly cold tolerant wild crape myrtle in Thompson seedless grapes improved its cold tolerance, and the damage of the leaves of the transgenic strain was significantly lower than that of the wild-type leaves after cold stress at 0 °C (Shu et al. 2021). *VvBAP1* improves grapevine cold tolerance by regulating and controlling sugar content and activating antioxidant enzyme activity. It also improves the stability of the cell membrane and further enhances the cold tolerance of overexpressed ectopic plants (Hou et al. 2018). In addition, *VaMYC2* can positively regulate the cold tolerance of grapes by interacting with *VaJAZ1* and *VaJAZ7*, while activating the expressions of *VaCBF1* and *VaP5CS* (Hu et al. 2022). Furthermore, the expression of *RAPD* markers in 83 grape germplasms with different cold resistance and in three different hybrid combinations from the F1 generation linked the molecular markers *S241-717* and *S238-854* to the cold resistance genes of Chinese wild grapes (Zhang et al. 2010).

The above results indicate that researchers have identified many valuable cold resistance genes and important transcription factors of stress resistance in grapes. However, the relationship between these genes is still unclear, and further studies are needed to determine which transcription factors regulate these genes, whether they interact with cold signaling pathways, and the detailed mechanisms of these genes involved in cold stress processes, which will be of great significance for further studies on the function and mechanism of cold resistance genes in grapevines. In addition, the current molecular breeding research of cold-resistant grapes is still in the stage of identifying molecular markers, transcription factors and genes related to cold resistance, and the breeding of new varieties is still in the process.

### Cold resistant cultivation models

In recent years, researchers have carried out extensive and in-depth research on cold-resistant cultivation models and found that the selection of cold-resistant rootstock for seedling grafting, deep-drain cultivation, winter short shoot pruning, strengthening fertiliser management, sealing frozen watering, and cultivating new grape racks can significantly enhance tree vigour. The section mainly introduces to the benefits and drawbacks of cold resistant rootstocks and Crawled cordon mode (CCM).

#### Cold-resistant rootstock

**Fig. 5:**
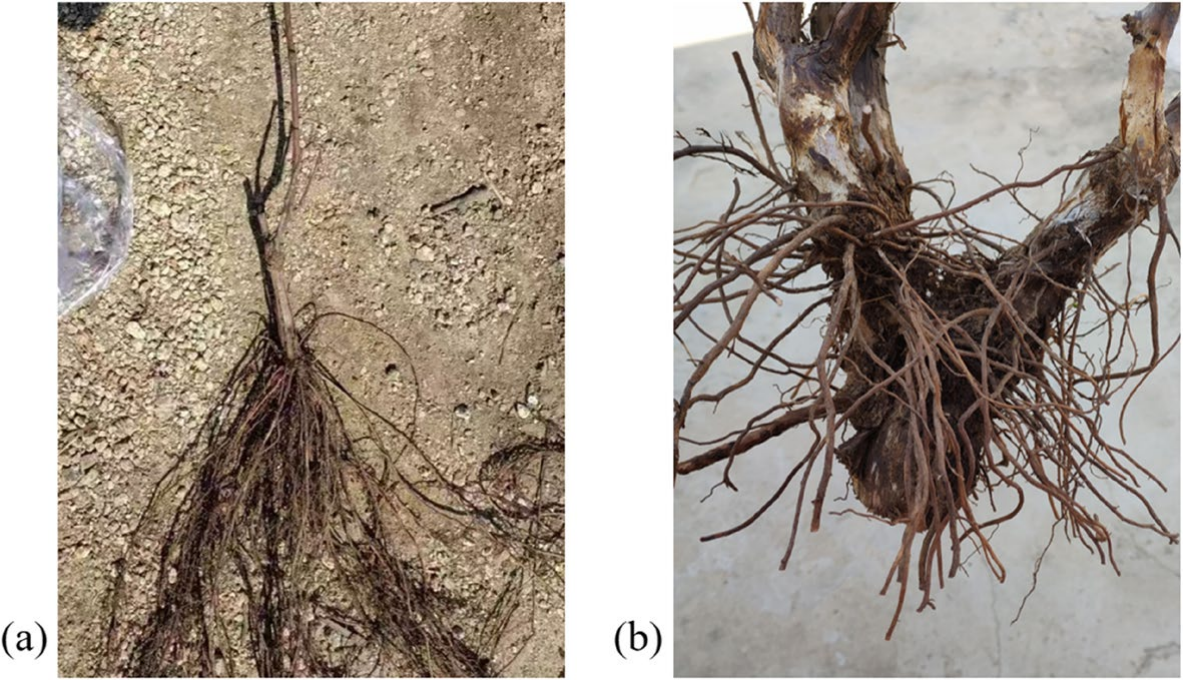
Comparison of root systems of *Beta* (**a**) and *Cabernet Sauvignon* (**b**)

Cold-hardy rootstocks could be an option for winter cold-challenged sites (Davenport et al. 2008). Targeted selection of rootstocks for grafting cultivation can not only maintain the good characteristics of the varieties, but also improve the adaptability and resistance to stress of good varieties, expand the range of cultivation range, and increase the yield (Liu et al. 2023). Additionally, it can reduce the thickness and width of the cover soil and reduce the cost of cold protection. At the same time, stock varieties should be selected according to the climatic and soil characteristics of the garden, the affinity of the rootstock and the scion, and tree potential. Local rootstocks with high yield, stable yield, high quality, and cold resistance, such as *3306C*, *3309C*, *5A*, *110R*, *Beta* (Fig. 5a), *LDP-191*, *LDP-294* (Zhang et al. 2009), etc., that have been successfully cultivated for many years should be selected. Furthermore, the study found that, under the same temperature, vines with deep roots are more resistant to cold, so it is necessary to avoid rootstocks whose roots are distributed in the surface layer (Gao et al. 2014).

However, grafted seedlings in the early planting stage are prone to various problems, such as the twigs being easy to break. Furthermore, in areas with sticky soil texture, high groundwater levels, high pH value, high salt content, and poor drainage, the grafted seedlings that used *Beta* (*V. labrusca* × *V. riparia*) as rootstock were prone to iron deficiency and yellowing (Yang et al. 2020). And in the arid and semi-arid areas of northwest China, *Beta* may not be the most suitable rootstock due to its sensitivity to drought (Magro et al. 2016).

When we perform crossbreeding to generate genetic variability, we can find different levels of incompatibility between the cultivar and the rootstock that need to be specifically evaluated. Also, studies show that there is no universal stock due to regional and variety diversity (Li et al. 2019a, b). Therefore, its ability to survive and produce in cultivation areas outside the breeding site needs to be verified (Lo Ay and Doaa, 2020). Further, rootstock breeding programs have high potential to adapt to grape growing challenges. More genetic studies on stocks to improve resistance to low temperatures are warranted. Rootstock-breeding programs have been scarce in recent decades, and most of the rootstocks used today were bred a century ago, when the needs of the sector were very different from today. Only eight organizations have released new successful rootstocks to the market during the twenty-first century (Marín et al. 2023). But this also shows the complexity of rootstock breeding.

#### Crawled cordon mode (CCM)

**Fig. 6:**
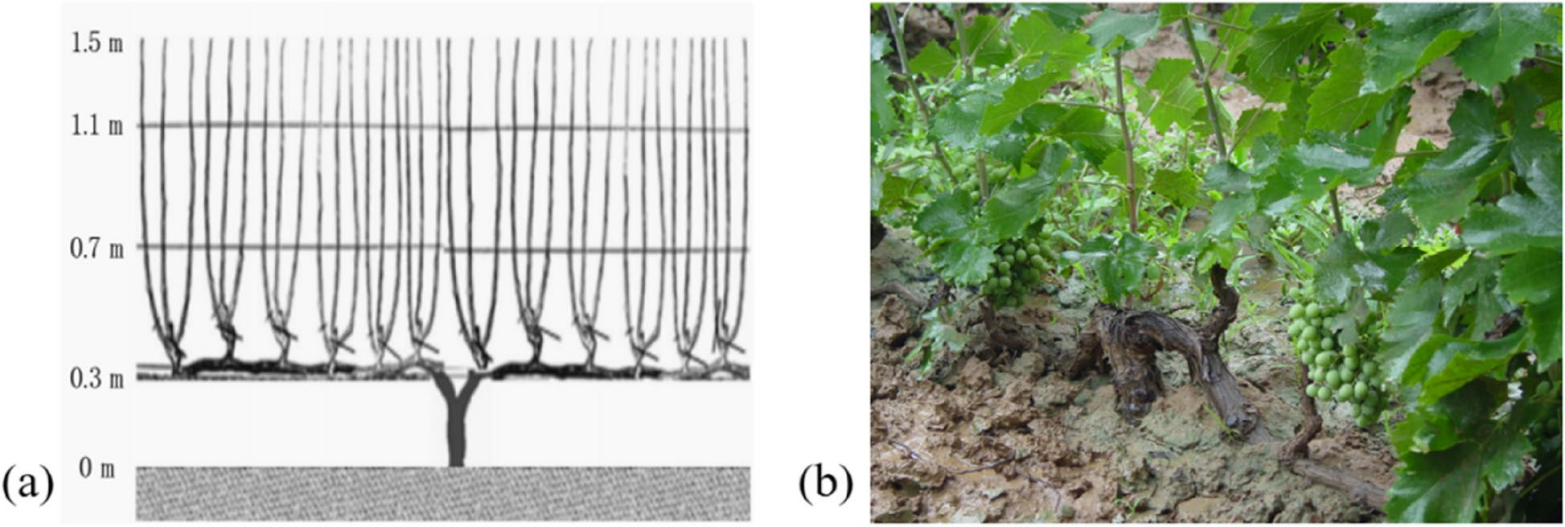
Mechanism (**a**) and image (**b**) of CCM

Li Hua et al. developed a new type of grape rack, the crawled cordon mode (CCM), in response to the cumbersome problems of burying vines with soil in winter and shelving them in spring (Fig. 6) (Li and Wang 2018). In this model, the trunk was lowered and the one year branches or caudexes were fixed on the first wire 30–40 cm above the ground, and the new branches were tied vertically to the shelf during the growing season. After pruning the short shoot, directly cover the main vine with soil or quilts.

Although the cultivation model cannot completely remove soil, it can improve the efficiency of removing soil in winter. CCM can reduce the intensity of labour in vineyard management, saving time and cost. It is conducive to grape quality and stable yield. The lower fruiting area gives the grapes nearly equal in ripeness (Zhang et al. 2021). At the same time, after winter pruning, the pruning left on the shelf forms wind barriers, which prevent wind erosion, blowing sand and ecological damage in winter, reduce the amount of erosion by 75.85% (Zhang et al. 2021), and improve the ornamental value of the vineyard. In addition, growing grass between lines can increase the content of aroma compounds, present more floral scents and some sweet and ripe fruit scents, improve wine quality (Xi et al. 2011), and significantly increase soil organic matter content (Griesser et al. 2022). Furthermore, the study showed that in Shanxi province, the overwintering cost of CCM was 3,300 yuan per hectare (Zhang et al. 2013a, b). Compared with the common fan-shaped method of multi-main vine, the cost of grape winter shearing, soil burial, and unearthing vines was significantly reduced.

However, under this cultivation model, a large number of fibrous roots and grape fruits are close to the ground, which are easily contaminated by soil (Han et al. 2021). In general, CCM is suitable for popularisation in cold viticultural regions.

### Physical and chemical methods

Physical cold prevention is a kind of cold prevention method that is widely used today. It mainly includes covering insulation material, spraying liquid mulch, spraying water in the orchard before frost, using night smoke, blowing, and other physical methods. At present, the common method of chemical cold prevention is spraying exogenous antifreeze (inorganic salt antifreeze, organic compound antifreeze, and plant hormone antifreeze) to enhance the resistance to cold in tree bodies. However, methods of smoking at night and spraying water on orchards before frost damage the environment and waste resources. The following mainly introduces four covering materials and exogenous antifreeze.

#### Covering material

Mulch cultivation technology is a kind of farmland planting technology that uses different heat insulating materials to cover the surface of the field to keep warm, preserve moisture, and prevent weed growth. Some previous studies mainly focused on comparing the thermal insulation effects of different materials, and the materials used were mostly adapted to local conditions, such as straw and branches (Prosdocimi et al. 2016). Although these materials are convenient and easy to obtain, it is difficult to ensure the repeatability of the cold protection effect because of the lack of unified technical parameters. Currently, the main covering materials on the market are plastic film, cold-proof cloth, insulation quilts, and liquid mulch, which have the advantages of simple operation and cost reduction. It has been applied in many severe-cold viticultural regions (Li et al. 2021a, b).

**Fig. 7:**
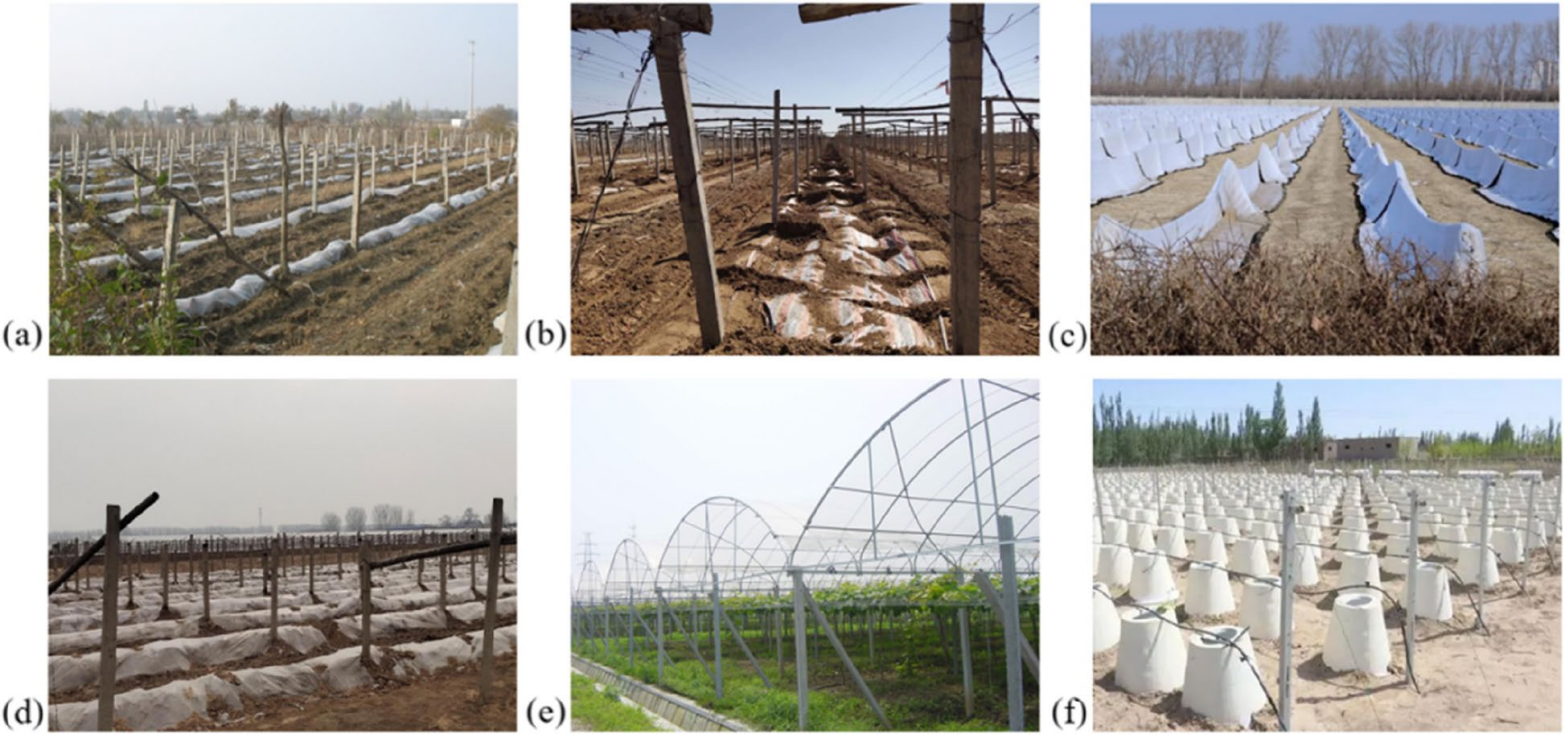
Image of four cover materials, greenhouse and vertical protective capsule. **a** Plastic film; **b** Cold-proof cloth; **c** Insulation quilts; **d** Liquid mulch; **e** Greenhouse; **f** Vertical protective capsule

1) The plastic film mulching method is a kind of cultivation technology for resisting drought and conserving soil moisture that can improve soil water and heat characteristics, regulate temperature, and expand crop planting areas through film evaporation inhibition and rain collection (Gao et al. 2019) (Fig. 7a). Before covering the plastic film, the soil should be leveled, the grape branches should be pressed close to the ground and covered with straw. In addition, a thin layer of soil should be pressed around it. On the basis of plastic film mulching technology, a double film mulching technology was invented which the middle of the two layers of film was raised to mimic the thermal insulation effect of double-layer glass windows in northern buildings (Li et al. 2018). The ground temperature after mulching in winter was significantly higher than that of covering the vines with soil, which could effectively prevent root frostbite, improve the survival rate of self-rooted seedlings, reduce the degree of freezing of branches and vines, increase the growth period of grapes, facilitate increases in grape yields, and avoid a large amount of water loss from branches and vines (Gutierrez-Gamboa et al. 2021; Li et al. 2011a, b).

However, today, the plastic films used in China are mainly EVA, PVC, PE, and PVC. Their half-life in the environment is very long and highly uncertain, and it may take hundreds of years to completely degrade (Li et al. 2021a, b). The amount of plastic film left on farmland grows year by year, seriously damaging soil structure and reducing water retention capacity (Chah et al. 2022). It is not conducive to the absorption of soil water and nutrients by the crop. Increasing plastic loads can lead to long-term changes in soil properties, such as water retention capacity, microbial activity and diversity, nutrient availability, and soil structure (MacLeod et al. 2021; Ren et al. 2023). In addition, as the plastic film deteriorates and breaks, a large number of agricultural film residues enter the soil, resulting in agricultural "white pollution" (Rhodes 2019; Zhang 2006). Therefore, it is necessary to rationally use plastic film mulching technology and strengthen the prevention and control of residual plastic film.

2) The cold-proof cloth mulching method is to lay the cloth directly on the first iron wire after winter shear, mulch all the grape branches, and press the ground on both sides with soil (Fig. 7b). Compared to covering vines with soil, this method plays a better role in soil moisture preservation, improves above-ground and underground temperatures effectively, and reduces the damage to grapevines (Niu et al. 2021). Furthermore, cloth can be manually or mechanically recycled and reused for many years, and storage is relatively simple compared to the straw, quilts, or other materials (Sun et al. 2015). However, under the cold-proof cloth mulching measure, the germination rate of the grape is significantly reduced and the vigour of growth is weaker than that covered with soil (Li et al. 2021a, b). However, the recovery of cold-proof cloth needs more labour and a high-cost, high-effect, low-cost machine, which requires extensive research.

3) The insulation quilt mulching method is to directly cover vines with insulation quilts in winter and unveiled in the next March. The specific thickness depends on the actual regional climate conditions (Fig. 7c). Compared to the traditional method, mulching insulation quilts not only increase the ground temperature but also increases the germination rate and fruit branch rate of grapes, which increases the planting income. At the same time, based on the 20-year use period, the annual production cost of insulation quilts is equivalent to 2.20 per meter, which is about 1/3 of the wear of the covering with soil. However, the study showed that although the use of insulation quilts can allow grapes to safely overwinter in Xinjiang, risks still exist (Li et al. 2014). Currently, the use of insulation quilts mulching method is gradually being accepted by large-scale grape production enterprises and shows a strong momentum of development.

4) Liquid mulching film is used to cover crops with a layer of humic acid macromolecular substances and humic acid suspension agents, coagulants, and other nutrients that can be absorbed by crops to form a layer of multimolecular mesh film that closes plant surface pores of the plant, reduces water evaporation, but does not affect water absorption (Fig. 7d).

Intensive research in recent decades has led to the commercialization of several bioplastics with industrial value, such as polylactic acid (PLA) (Yu et al. 2023), polyhydroxyalkylate (PHA) (Corre et al. 2012), polybutylene succinate (PBS), and thermoplastic starch (TPS), as well as biopolyethylene terephthalate (bioPET) and biopolyethylene (bioPE). The degradation of PBS, PBS starch, and PLA bioplastics did not affect bacterial diversity in the soil environment, but the degradation rate was low in the soil (Mukherjee et al. 2023). Similarly, BLF can promote the accumulation of sugars and polyphenols, improve the overall quality of grapes (Xue et al. 2019), and gradually degrade naturally to organic humic acid fertilizers through light, heat, and microorganisms in the soil in about 70 to 90 days, avoiding environmental pollution. Furthermore, the application of hydrophobic granular film (CM-96–018) has reduced the freezing damage of potato, grape, and citrus (Fuller et al. 2003) and has considerable anti-freezing protection prospects (Centinari et al. 2016).

Biodegradable films have better mechanical properties, heat resistance, and moisture resistance (De et al. 2021), are reusable, increase yield, and reduce mortality (Buesa et al. 2021), and show significant improvements in soil conditions and water use efficiency (Zhou et al. 2021a, b, c). At the same time, it takes less time than covering vines, preventing delays in grape production due to labor constraints (Luo et al. 2010). And it is an eco-friendly production mode that will not damage the soil surface, will not produce sand-blowing weather under strong winds, and can also reduce the use of plastic film.

Overall, the most widely used products in China are still plastic film, cold cloth, and insulation quilt covers. Although liquid mulch has received a great deal of attention due to its biodegradability and has been studied for nearly a century, its widespread industrialization is still in its infancy, and its applications are limited (Fredi and Dorigato 2021). On the one hand, the cost is too high, which is difficult to bear in production (Li et al. 2022), and, on the other hand, the effect of cold prevention is unstable from year to year, which has certain risks (Li et al. 2013). In the severely cold areas of northern China, the existing liquid mulch is not sufficient to prevent root systems from freezing, and it cannot be popularized in a large area (Liu et al. 2022a, b).

#### Exogenous antifreeze

Currently, of spraying exogenous antifreeze is the most common method of chemical cold prevention, which can improve cell fluid, enhance plant frost resistance, and significantly reduce the degree of frost damage to grape leaves (Song 2016). Compared to cultivation measures, antifreeze has the characteristics of acute response, high efficiency, simplicity, and low cost (Shi et al. 2014). In recent years, the research on exogenous antifreeze has gradually increased, and a variety of effective plant antifreeze agents have been successfully screened out, which are mainly divided into inorganic salts (calcium chloride), organic compounds (salicylic acid (Ma and Ma 2016), aminobutyric acid, naphthalene acetic acid), plant hormones (brassinolactone, abscisic acid, melatonin), and new plant antifreeze agents (chloranilide, malehydrazide, etc.) (Liu et al. 2022a, b). Studies have shown that potassium dihydrogen phosphate & brassinolide" is a cost-effective combination of antifreezing that can adjust the endogenous hormone balance of crops, enhance resistance, alleviate drug damage, help crops recover quickly, and significantly reduce freezing damage (Li et al. 2021a, b).

However, plant antifreeze can only be used in the edge of areas that need to be covered with soil. In extreme long-term cold conditions, it is difficult to achieve good antifreeze effect. At present, the effect of antifreeze is unsatisfying and the cost is high, which can only be used as an auxiliary method. Besides, there is a lack of effective data on the difference in the effect of plant antifreeze among different grape varieties, and the effect of different plant antifreeze on the cold resistance of the same grape variety needs to be further investigated. The evaluation of the effect of some antifreeze agents, as well as the mechanism of action, is incomplete. In the future, attention should be paid to the research and development of multicompound plant antifreeze to improve the cold resistance of grapevines. Develop low-cost, nontoxic, no residue, fast-degrading plant antifreeze in order to save economic costs and improve grape production efficiency.

### Grape protected facilities

Grape facility cultivation uses specific facilities to improve or control the environmental factors that affect grape growth and development in open soil in the cold-resistant areas that are not suitable for grape growth, so as to artificially create an environment suitable for crop growth. Therefore, the crop can fully mature, accumulate more nutrients, lay a material foundation for safe overwintering and a high yield in the next year, and achieve the goal of high quality, high yield, and stable yield (Pang et al. 2019). The following mainly introduces the advantages and disadvantages of greenhouses and protective warehouses.

#### Greenhouse

The greenhouse is built with thermal insulation, moisturising, light, and other equipment and technology to protect thermophilic plants from the cold, or to promote growth, early flowering, and fruiting (Fig. 7e). It can be classified as multi-span greenhouses, solar greenhouses, plastic greenhouses, small arch sheds, breeding sheds, and new cold-proof equipment. The solar greenhouse and the plastic greenhouse are the most widely used in the cultivation of fruit trees in the north of China. The plastic greenhouse is easy to construct, convenient to use and relatively low-investment, which makes it more suitable for less developed area in economic (Zhao 2017).

Compared to burying with soil overwintering, greenhouses artificially adjust moisture and temperature (Khoshnevisan et al. 2013) so that the grape branches and roots can basically overwinter safely, and it is not easy to dry. It can greatly extend the growth period, increase the effective accumulated temperature, and overcome adverse factors such as the short frost-free period and absolute low temperature, so that plenty of superior varieties can be the main varieties in the alpine region and north China.

However, in the face of extreme weather phenomena, its insulation, moisturising effect, and firmness remain to be verified. At the same time, greenhouse cultivation reduces the ability to supply mineral nitrogen to the soil, requiring more nitrogen fertilizer to ensure rapid growth of vegetables in the greenhouse (Dan et al. 2022). In addition, leaving aside the high cost of the initial investment, labour and energy typically account for over 50% of operating costs in smaller glasshouse units (Lin et al. 2022). Setting up a test shed of 50 square metres requires an investment of ￥20,000, and the production cost is higher than that of open-air cultivation (Ozkan et al. 2007). However, most of our wine grape varieties have a low unit price and output value, so it is not economical and practical to set up greenhouses during the winter. Furthermore, the adoption of greenhouse cultivation has an impact on subsequent mechanized fertilization, harvesting, and other steps and can only choose manual operation, which reduces work efficiency and increases the cost (Mu et al. 2014).

#### Protective capsule

The protective capsule (Fig. 7f) is an effective wintering method proposed by Li et al. (2019a, b), in which the protective silo is attached to the tree before overwintering, after the body of the tree has been clipped, for drug control, garden clearing, and winter irrigation. The use of a protective capsule opens up a new way of wintering grapes and achieves a win–win situation for tree body protection and ecological cultivation. On the one hand, the mode is more beneficial for ensuring yield during the growing period, and tree body shaping (Zhang et al. 2019). On the other hand, this technology no longer changes the soil between the grape rows to bury in winter and remove in spring, thus realising a new ecological cultivation mode of planting grass or natural grass between the grape rows, which avoids the traditional disadvantages of covering vines with soil and destroying ecology (Li et al. 2019a, b). Furthermore, as the protective capsule is light to cover and recover, it saves time and labor. However, since each vine must be held, the required number of protective capsules is large and the initial input cost is high. In addition, the recovery and storage of the protective capsule after its removal each year requires a certain amount of manpower and warehouse space.

Similarly, a grape overwinter insulation facility is proposed, which consists of two parts: a base cover made of hard insulation material and a temperature control sleeve made of composite material (Liu et al. 2018). The base cover is attached to the ground along the grape planting line and fixed with pins. The temperature-regulating sleeve is placed on the base cover and fixed to it. The corresponding temperature-regulating sleeve is configured in different regions. The cold-proof facility has a wide application area, a good cold-proof and insulation effect, high working efficiency, simple installation and removal operations, and can be used for many years. The experiment demonstrated that the facility met the temperature requirements for grape safety during winter and spring germination. However, the weather conditions in the cold region are changeable, so it is necessary to do further research and experiment with the materials and parameters of the temperature control jacket.

## Prospect

There are still some challenges to be solved in the development of overwintering measures that prevent burial in China. First, although great progress has been made in breeding for cold resistance, it was found that the resistance of new varieties and the quality of the wine could not be combined, and no high-quality cold-resistant varieties that could be widely promoted. At the same time, the relationship between the labeled cold-resistance genes remains unclear, and it is still necessary to determine which transcription factors regulate these genes and the detailed mechanism of these genes involved in the cold stress process, which is of great significance for further study of the function and mechanism of cold-resistance genes in grapevine. Second, the compatibility between the hardy rootstock and grape varieties and whether they can survive and produce in the cultivation area outside the breeding area need further research. Third, the effectiveness of newly developed coating and anti-freezing materials, degradable liquid film, and external antifreeze in practical applications remains unknown, as does whether it will cause additional soil pollution. Finally, although grape-protected cultivation can effectively solve cold resistance, large-scale facilities will bring huge construction and maintenance costs, and often leads to more serious diseases. Therefore, at present, these new wintering measures are not enough to replace the cover of vines with soil in northwest China for comprehensive promotion.

Furthermore, with global warming, the Chinese buried cold protection line keeps moving northward (You 2007), which makes the suitable areas to grow wine grapes expand in northern China, and it is likely that some potential high-quality wine grape production areas will appear. At the same time, buried soil for cold protection is no longer necessary in some northern areas, which is conducive to the economic and safe wintering of grapevines, which reduces production costs. Optimizing the layout of the grape industry is of great importance. However, the threat of extreme low temperatures to grapevines in winter remains objective. In order to ensure the sustainable development of wine grape production, the Chinese grape industry should accelerate the transformation of production modes, accelerate the process of agricultural mechanization, and further use eco-friendly technology to replace the overwintering measure which covers vines with soil, so as to protect the diversity of viticulture and related ecosystems. The future work of the Chinese grape industry will focus on the transition from traditional cultivation to labor-saving, and mechanized directions, and the Chinese grape industry will undoubtedly develop rapidly in high-quality, green, efficient, and sustainable directions.

## Conclusion

Overall, soil-burying remains the most common and effective method to safely over-winter in northern China. Nevertheless, it damages the ecological environment, costs more and requires a lot of labour. As alternatives, novel grape breeding, cultivation models, covering materials, and protected grape facilities could effectively improve the economic benefits of grape production and minimize the damage to the soil surface, but their practical application in vineyards in northern China is still limited due to the unstable insulation effect from year to year. Moreover, some wintering technologies might face other challenges, such as the resistance of new varieties and the quality of wine does not combine, and large facilities bring more serious diseases. In the future, numerous studies are needed on overwintering measures which avoiding burial, especially mechanized, labor-saving and environment-friendly technologies, to transform the traditional production mode. It is believed that, in the future, the safety of overwintering measures which avoiding burial will be improved, and the cost of wineries will be reduced, thus benefiting consumers.

## Data Availability

Yes.
